# Intensification of common bean and maize production through rotations to improve food security for smallholder farmers

**DOI:** 10.1016/j.jafr.2020.100040

**Published:** 2020-12

**Authors:** Eliakira Kisetu Nassary, Frederick Baijukya, Patrick Alois Ndakidemi

**Affiliations:** a School of Life Sciences and Bioengineering, The Nelson Mandela African Institution of Science and Technology (NM-AIST), P.O. Box 447, Arusha, Tanzania; b International Institute of Tropical Agriculture (IITA), P.O. Box 34441, Dar Es Salaam, Tanzania

**Keywords:** Food crops, Smallholder farms, Sustainable cropping systems, Tanzania

## Abstract

A field experiment was conducted to understand whether non-formalized monocultures of maize could be substituted by the rotations with common bean on smallholder farms. This study was installed in the northern highlands of Tanzania along the slopes of the highest African peak of Mt. Kilimanjaro with the predominance of smallholder farmers. Cropping seasons (S), cropping systems (C), bean varieties (V), and their interactions were evaluated. Data collected were plant height, ground coverage, total biomass, number of pods per bean and seeds per pod, 100-seed weight, and grain yield. Results indicated that bean in long rainy seasons produced significantly larger grain yields as an effect of S (3.3 t ha^−1^) in 2015, C (3.4 t ha^−1^) in intercrop, V (2.7 t ha^−1^) in local bean, S × C (4.4 t ha^−1^) in 2015 in intercrop, S × V (3.4 t ha^−1^) in improved bean in 2015, C × V (4.6 t ha^−1^) in intercropped local bean, and S × C × V (5.0 t ha^−1^) in intercropped local bean in 2017. In a short rainy season, significantly larger bean grain yield (1.8 t ha^−1^) was recorded as an effect of C when sown subsquent to maize. The effects of V and/or C × V were not significant on bean grain yield during short rainy season. Maize in long rainy seasons produced significantly larger grain yields as an effect of C (2.9 t ha^−1^) but not for S and S × C in rotation with the local bean. In short rainy seasons, significantly larger maize grain yield was produced in 2015 (2.6 t ha^−1^) but the effects of C and S × C were not significant in 2015 and 2016. This study concluded that inclusion of intercrops (of maize and common bean) as part of a rotation with one of these crops significantly improved grain yields and hence provided promising grounds of the options for sustainable food production on smallholder farms.

## Highlights

• Rotation of maize and common bean could improve food security of smallholders.
• Cropping seasons, practices and bean varieties interactions improved crop yields.
• The highest bean yield (5.0 t ha^−1^) was obtained as an effect of these interactions.
• Higher bean yield (1.8 t ha^−1^) was obtained in bean sown as a subsequent to maize.
• Higher maize yield (2.9 t ha^−1^) was obtained in a rotation with the local bean.

## Graphical abstract

**Figure:**
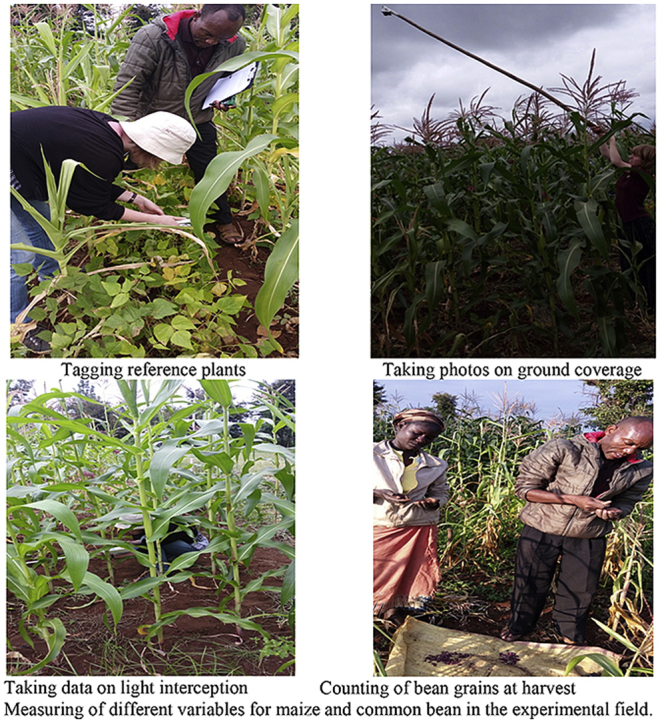

## 1 Introduction

Maize (*Zea mays* L.) is the most important cereal crop for food and cash in Sub-Saharan Africa (SSA), Asia and Latin America [1]. Maize is produced throughout the world, with the United States, China, and Brazil being the top three producing countries [1]. Maize accounts for 30–50% of low-income household expenditures containing starch (72%), protein (10%), fat (4%), and energy density of 365 Kcal/100 g [2]. Of the worldwide maize consumption as food, Africa consumes most (30%) of its production and the highest (21%) is in SSA [[1], [2], [3]]. However, the global consumption of maize is expected to increase by 16% by 2027 as animal feed and for human consumption due to the expanding livestock sector and population growth [4]. Therefore, deploying practices of increasing food production through sustainable intensification of agricultural systems in densely populated smallholder settings could be an important option.

Common bean (*Phaseolus vulgaris* L.) is the most produced and consumed food grain legume worldwide, with a market value exceeding all other legumes [[5], [6], [7]]. Common bean is cultivated mainly for subsistence as a major source of dietary protein of smallholders and consumed mostly on-farm, so it is difficult to accurately estimate total global production [8]. There is always an overestimation of the total area planted and underestimation of global yields of common bean due to the widespread practice of its production through intercropping with cereals [9]. Common bean has the potential of reducing poverty and increase food security on smallholder farms [10] if someconstraints to production are addressed. Smallholder farmers depend largely on maize and common bean as important sources of food and income [[11], [12], [13]]. Despite the importance of maize and common bean, their yields have often remained very low (≤0.5 t ha^−1^) relative to a potential of 1.0–3.5 t ha^−1^ for common bean [7,14,15]. The yields for maize are 0.5–1.5 t ha^−1^ against a potential of 1.5–6 t ha^−1^ often recorded on smallholder farms [16]. The low yields of maize and common bean are attributed to the poor soil fertility with little or no use of fertilizers, continuous use of varieties with low genetic potential and high incidences of diseases and pests [[16], [17], [18]].

A crop rotation farming where N_2_-fixing legumes are involved with a non-N_2_-fixing crop in the same piece of land is known to increase productivity. The productivity of a subsequent crop after N_2_-fixing legume is often optimized by the additional benefits contributed by the N_2_-fixer crop [[19], [20], [21]]. The productivity of cereals in rotation with legumes is largely due to the improvement of soil fertility, minimization of diseases and pests and the influence of other rotational effects [22,23]. However, legumes contribute poorly to the improvement of soil fertility through N_2_-fixation in adverse environmental conditions [24]. On the other hand, rotational cropping may be risky to the farmers in situations where a single crop in the season fails to attain the yield production stage [25].

Rotation of cereals/maize with grain legumes/common bean is one of the important elements of sustainable intensification in highly populated areas due to continuous reduction in cultivated land [26]. However, there is limited information about the appropriate options by which these rotations may be practiced in a given cropping season (short or long rainy seasons) and the varieties of common bean (local or improved) cultivated by smallholder farmers [15]. Considering the agronomic importance of cultivating common bean including residual effects on the subsequent non-N_2_ fixing crops, it is important to understand the benefits derived from different varieties of common bean on the system productivity. Smallholder farmers often cultivate local varieties of the common bean as part of a rotation with maize [15,27,28]. Also, apart from the continuous use of these local varieties, there are still options for the inclusion of improved varieties, which are high yielding [15,25]. The local and improved varieties of common bean could be practically compared for their benefits on the subsequent maize crop and the overall return to the farmer on smallholder systems. Therefore, this study focused on assessing the productivity of maize and determinate (improved) and indeterminate (local) varieties of the common bean by understanding whether non-formalized monocultures of maize during long rainy seasons could be substituted by the rotations with common bean and close the gap associated with low yields of these crops on smallholder farms.

## 2 Materials and methods

### 2.1 Description of the study site

**Fig. 1:**
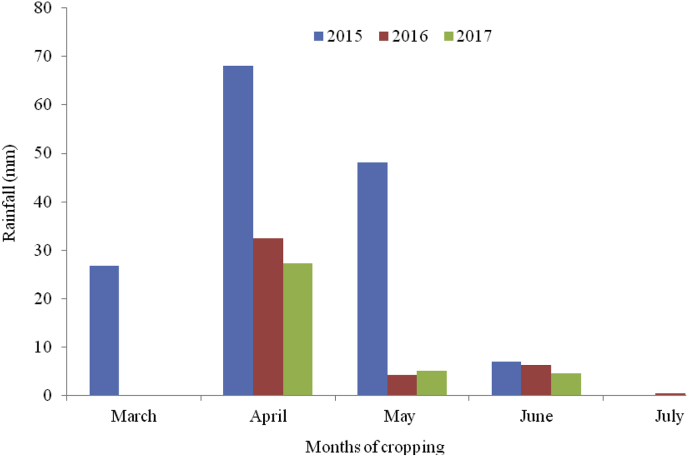
Trends of the mean monthly rainfall in the experimental site during the growth periods of the crop in 2015, 2016, and 2017 long rainy seasons.

An experiment was based on field work in the Kilimanjaro region representing the northern highlands of Tanzania. Mt. Kilimanjaro is the highest peak (5895 m) of the cultivated highlands in Africa although the cropped land ranges from below 900 to 1800 m above sea level. The location of the established field is 03°18′03.74″ S and 37°12′13.94′ E and the altitude is 1051 m above sea level [28,29]. Rainfall ranges between 800 and 1200 mm annually [27]. Rainfall trends through the growing periods of crops (2015 to 2017) in the experimental field are presented in Fig. 1. The soils are volcanic ash in parent materials, highly weathered and infertile [27,30,31]. However, before the establishment of experimentations in 2015, basic soil characterization was conducted and the results are presented in Table 1.

**Table 1:** Chemical properties of the soils in the study area before the installation of experimentation.

Parameter	SI–Unit	Value	Rating category [27]
pH_(H2O)_		6.02	Medium acid (5.6–6.0)
Available phosphorus (P)	mg kg^−1^	5.8	High (>25)
Exchangeable bases:	cmol_(+)_ kg^−1^		
- Potassium (K)		0.27	Low (0.20–0.40)
- Sodium (Na)		0.62	Medium (0.31–0.70)
- Calcium (Ca)		8.00	Very high (>5.0)
- Magnesium (Mg)		0.53	Low (0.3–1.0)
Total nitrogen (N) (%)		0.12	Low (0.1–0.2)
Organic carbon (%)		1.79	Very low (<2)
Organic matter (%)		3.09	Low (3–7)
C/N ratio		15:1	Medium (10–15)
Micronutrients:	mg kg^−1^		
- Zinc (Zn)		1.42	High (>1)
- Iron (Fe)		38.33	High (>4.5)
- Manganese (Mn)		35.22	High (>1)
- Copper (Cu)		0.18	Low (deficient) (0–0.4)

### 2.2 Experimental design and treatments

This experiment involved long and short rainy seasons from 2015 to 2017. A randomized complete block design (RCBD) of assigning treatments to experimental plots was used. Table 2 presents a summary of treatments used in each cropping season. In each long rainy season the treatments were: (1) monocultures: (i) three levels of maize (M); (ii) improved bean (IB); (iii) local bean (LB); and (2) intercrops: (i) maize with improved bean (M+IB); (ii) maize with local bean (M+LB). Since rotational effects were the main objectives of this study, the strategy was met by introducing a sequence of rotations in the first short rainy season but the design was based on the very first long rainy season of 2015. Therefore, the treatments in each short rainy season were: (1) five levels of monoculture maize (M); (2) two levels of monoculture improved bean (IB); and (3) two levels of monoculture local bean (LB). All treatments and/or some in their respective levels in each cropping season were in four replications. The installation of experimentations in the 2015 long rainy season was the establishment of the study; so, the basis of the treatments shown in Table 2 was not expected to be IB (long) ​+ ​LB (short), or LB (long) ​+ ​LB (short). The improved bean variety *Lyamungu 90* bred for higher yielding was obtained from Selian Agricultural Research Institute based in Arusha Tanzania while the local bean variety *Mkanamna* was sourced from the local markets. Maize variety Dekalb brand DK8031 was used throughout the experiment. Both bean varieties were included in all cropping seasons as also smallholder farmers often do not have the exact choice of a certain bean type to be cultivated in rotation (as a monocrop) or as part of an intercrop with maize. So, it was important in the present study to test the performance of rotations with maize and varieties of common bean under each cropping season (long and short).

**Table 2:** Indication of treatments as used in the field experiments. Treatments were replicated four times at every cropping season.

Years of cropping, rainy seasons, and treatments				
2015		2016		2017
Long	Short	Long	Short	Long
M	M	M	M	M
IB	M	IB	M	IB
LB	M	LB	M	LB
M ​+ ​IB	M	M ​+ ​IB	M	M ​+ ​IB
IB	IB	IB	IB	IB
M ​+ ​LB	M	M ​+ ​LB	M	M ​+ ​LB
M	LB	M	LB	M
M	IB	M	IB	M
LB	LB	LB	LB	LB

**Key:** M = Maize, IB = improved bean, LB = local bean.

### 2.3 Experimentation

Each experimental plot was 5 m × 3.2 m in size with a path of 1 m between replicates and 0.5 m between treatments. The germination test was over 98% for all maize and bean seeds. The sowing spacing for maize seed was 80 cm between rows and 30 cm between plants. Bean seed was sown at a spacing of 40 cm between rows and 10 cm between plants in a row. However, in intercrops of maize with the beans, the beans were sown at a distance of 40 cm from the two maize rows. Two seeds of each crop type were sown in a hole and thinned to one seedling at 14 days after sowing. In a monoculture bean and that intercropped with the maize, the plant populations were 286,875 and 127,500 bean plants/ha, respectively. For maize, the plant population was equivalent to 53,125 maize plants/ha. Plant nutrition management involved an application of P and N containing fertilizers at sowing and stages of plant growth, respectively. The source of P was triple superphosphate (TSP, 46% P_2_O_5_) applied to the planting hole at a rate of 25 kg P ha^−1^. When plants were 21 days after sowing, urea (46% N) was applied at a rate of 120 kg N ha^−1^ around each maize plant to contribute to N nutrition [32]. Removal of emerging weeds and the use of water for supplemental irrigation were done during the period of crop growth.

### 2.4 Data collection

The sowing of maize and bean was done simultaneously during each cropping season. However, the sowing and harvesting dates and months differed with the cropping seasons and type of the crop. In the 2015 long rainy season sowing was done on 26 March but common bean was harvested on 29 June and maize on 15 August. During the 2015 short rainy season sowing was done on 13 November but the bean and maize harvesting were on 9 February and 5 March 2016, respectively. In the 2016 long rainy season sowing was done on 30 April but the harvesting of bean and maize was on 25 July and 18 August, respectively. Sowing in the 2016 short rainy season was done on 5 November 2016 but only maize reached maturity and harvesting was done on 12 February 2017. Further, in the 2017 long rainy season sowing was done on 9 April 2017 but harvesting was on 15 July and 23 August bean and maize, respectively.

During data collection, plants of the inner rows in each plot were randomly identified and used for the measurements. The growth characteristics measured are the plant height and leaf canopy coverage on the ground when the plants were 42 and 56 days from sowing as beyond 56 days there was no further increase in these variables. A 1 m × 1 m quadrat frame was used for measuring ground coverage and the tape-measure was used for measuring plant height. Depending on the plant type in all plots, the side rows and the first three inner plants on both sides of a row were considered as guards and not included in the measurements. At harvest, the data collected from the same bean plants used for growth measurements were the total weight (stover and grains) for biomass determination, number of pods per individual bean plant (from 10 randomly selected plants), number of seeds per pod (from 50 randomly selected pods), weight of grains for yield determination, and weight of 100 seeds. The data collected for maize at harvest were the total weight (stover and grains) for determination of biomass yield, dry grain yield, and weight of 100 seeds. Total biomass yields and grain yields (t ha^−1^) were computed using the formulae 1 and 2.

(1) $$\text{Biomass}\;\text{yield}\;\left(\text{t}{\text{ha}}^{-1}\right)=\frac{Total\;weight\;\left(in\;tons\right)\times 10^{4}}{Actual\;harvest\;area\;\left(m^{2}\right)\;}$$

And,

(2) $$\text{Grain}\;\text{yield}\;\left(\text{t}{\text{ha}}^{-1}\right)=\frac{Grain\;weight\;\left(in\;tons\right)\times 10^{4}}{Actual\;harvest\;area\;\left(m^{2}\right)\;}$$

The actual harvest areas were 9.36 m^2^ for sole and maize intercropped with common bean, 10.92 m^2^ for common bean sown in monoculture, and 6.24 m^2^ for common bean intercropped with maize.

### 2.5 Statistical analyses

To isolate the effects of significant treatments F-test was used at a threshold of 5%. In analyzing data from beans cultivated during long rainy seasons the fixed effects were the cropping seasons (years), cropping systems, and bean varieties but in short rainy season (single) the fixed effects were the cropping systems and bean varieties whereas the replicates were treated as random factors. Analysis of the data collected from maize cultivated during the long and/or short rainy seasons involved treating cropping seasons and cropping systems as the fixed effects while the replicates were treated as random factors. For the beans in a short rainy season the main effects were the cropping systems and bean varieties. Maize was evaluated in both long and short seasons using cropping seasons and cropping systems as the fixed effects. The data was coded as bean-maize rotation and maize-bean rotation as testing of both could indicate an important question and hypotheses, that there could be a difference between lengths of rainy season and rotation (and interactions between year and rotation). This involved considering special contrasts or as beans and maize nested within monoculture and beans rotated/mixture times season was expected to yield more insight in the data and its interpretation. The use of season as the fixed effect is based on the observed variations of rainfall, its distribution and intensity in a specific season, which might not always be the same. Further, as the experiment was performed in a single location for five cropping seasons, the main effects and their interactions with location are confounded. Shapiro-Wilk test for normality of residuals and Bartlett's test for homogeneity of variances were performed in situations where main effects were not significant. The significance of effects is independent of the check of model assumptions. The mixed model approach is only valid if assumptions are fulfilled. In addition, multiple linear regression analysis was performed for grain yield as a response variate and the fitted terms being 100-seed weight, total biomass, seeds per pod, and ground coverage and plant height at weeks 6 and 8 to test relationships between grain yield and these variables. GenStat Discovery Edition 4 was used for all statistical analyses.

## 3 Results

### 3.1 Performance of common bean in long rains

The main effect of years of cropping was significant on all measured yield and yield components of common bean except the number of seeds per pod. Cropping systems were significant in affecting grain yield and all yield attributes of common bean but not on seeds per pod and 100-seed weight. The main effect of bean varieties on yield and yield related variables was significant except on total biomass. The interactions of cropping seasons, cropping systems, and bean varieties were significant on grain yield and 100-seed weight. The largest bean grain yield (5.0 t ha^−1^) was obtained in local bean intercropped with maize in 2017 cropping season while the largest 100-seed weight (56.28 g) was in improved bean intercropped with maize in 2016 cropping season. Significantly larger bean grain yield (4.4 t ha^−1^) was obtained in 2015 cropping season for beans intercropped with maize as interaction effects of cropping seasons and cropping systems. Similar significant interaction effects of cropping seasons and cropping systems were in 100-seed weight (40.25 g) where the larger weight was obtained in 2016 on plots which common bean started and ended during the years of experiment involved rotation with maize. The significantly larger bean grain yield (3.38 t ha^−1^) was obtained in improved bean in 2015 as effects of interaction between cropping seasons and bean varieties. Similar significant interaction effects were observed in total biomass (9.58 t ha^−1^) obtained in bean intercropped with maize in 2015 and 100-seed weight (55.08 g) recorded in improved bean in 2016. Further, a significantly larger grain yield (4.6 t ha^−1^) was obtained in local bean intercropped with maize as interactions of cropping systems and bean varieties (Table 3).

**Table 3:** Grain yield (t ha^−1^) and yield components including total biomass (t ha^−1^), number of pods per bean plant, number of seeds per pod, 100-seed weight, and yield of the common bean as affected by the long cropping seasons of years, bean varieties, cropping systems and their interactions.

Factors	Assessments	Measured variables in common bean				
		Yield (t ha^−1^)	Total biomass (t ha^−1^)	Pods per plant	Seeds per pod	100-seed wt (g)
Years of cropping (S)	2015 –Long rainy season	3.3b	8.8b	10b	2.7	34.6b
	2016 –Long rainy season	1.8a	3.6a	7a	3.2	39.8c
	2017 –Long rainy season	1.4a	2.1a	7a	2.9	31.6a

Cropping systems (C)	F&E (Rotation with maize)	1.5a	4.4a	6a	3.2b	35.1ab
	Intercrop with maize	3.4b	5.9b	10c	2.9ab	36.4b
	Monoculture	1.6a	4.2a	8b	2.7a	34.5a

Variety (V)	Improved bean *Lyamungu 90*	1.6a	4.4	5a	2.2a	48.4b
	Local bean *Mkanamna*	2.7b	5.3	11b	3.7b	22.3a

3-WAY-ANOVA (F-stat.)						
S		90.55 (*P* < .001)	45.14 (*P* < .001)	21.68 (*P* = 0.002)	0.63 (*P* = 0.562)	384.43 (*P* < .001)
C		70.14 (*P* < .001)	3.87 (*P* = 0.04)	5.12 (*P* = 0.017)	0.73 (*P* = 0.496)	0.77 (*P* = 0.480)
V		45.30 (*P* < .001)	1.52 (*P* = 0.228)	53.7 (*P* < 0.001)	28.28 (*P* < .001)	451.57 (*P* < .001)
S × C		9.38 (*P* < .001)	0.82 (*P* = 0.531)	1.01 (*P* = 0.429)	0.64 (*P* = 0.643)	3.25 (*P* = 0.036)
S × V		21.35 (*P* < .001)	4.42 (*P* = 0.022)	0.96 (*P* = 0.394)	0.06 (*P* = 0.938)	4.8 (*P* = 0.016)
C × V		20.25 (*P* < .001)	0.39 (*P* = 0.681)	0.53 (*P* = 0.596)	0.22 (*P* = 0.802)	3.06 (*P* = 0.064)
S × C × V		3.02 (*P* = 0.035)	1.84 (*P* = 0.151)	0.45 (*P* = 0.77)	0.26 (*P* = 0.901)	3.48 (*P* = 0.020)

Means with different letters differed significantly from each other. **Key:** F&E means common bean started (F =First) and ended (E = Ended) in the plot during the years of experiment involved rotation of common bean and maize. Means in a column for each measured variable bearing different letter(s) for each assessed treatment in a specific category of factors differ significantly. Main effect of bean variety on total biomass test statistic W = 0.9409 (*P* = 0.002); Chi-square = 0.00 on 1^0^ of freedom (*P* = 1.000). The main effect of years of cropping on the number of seeds per pod test statistic W = 0.9885 (*P* = 0.759); Chi-square is 4.76 on 2^0^ of freedom (*P* = 0.093).

Multiple linear regression analysis indicated that total biomass (*P* < .001) and the number of seeds per pod (*P* = 0.014) have a strong and significant influence on bean grain yield during the long rainy season. Also, the number of pods per plant, ground coverage, and plant height after six weeks had a positive contribution to grain yield although the influence was not significant (Table 4).

**Table 4:** Estimates of parameters generated from multiple linear regression analysis based on three long cropping seasons as their relationships with bean grain yield.

Parameter	estimate	s.e.	t(63)	t pr.
Constant (C)	−0.69	1.2	−0.57	0.568
100-seed weight (g)	−0.0092	0.0109	−0.85	0.401
Total biomass (t ha^−1^)	0.1681	0.0361	4.66	<.001
Pods per plant	0.044	0.036	1.22	0.226
Seeds per pod	0.2386	0.0947	2.52	0.014
Ground coverage (%) at week 6	0.0131	0.0226	0.58	0.563
Ground coverage (%) at week 8	0.0238	0.0272	0.88	0.384
Plant height (cm) at week 6	−0.0931	0.023	−4.05	<.001
Plant height (cm) at week 8	0.033	0.0223	1.48	0.145

The percentage variance accounted for is 65.3 and the standard error of observations is estimated to be 0.998. Weeks 6 and 8 represent 42 and 56 days, respectively after sowing.

### 3.2 Performance of common bean in short rains

Grain yield and yield attributes of common bean for the measurements taken in the 2015 short rainy season are presented in Table 5. The main effect of cropping systems was significant on grain yield while bean variety was significant on the number of pods per bean plant. Sowing of the bean as part of a rotation with maize in situations where maize started on the plot produced larger grain yield (1.8 t ha^−1^) compared with grain yield (1.7 t ha^−1^) obtained in bean sown as a monoculture. The main effect of bean varieties was significant on the number of pods per bean plant.

**Table 5:** Grain yield (t ha^−1^) and yield components including total biomass (t ha^−1^), number of pods per bean plant, number of seeds per pod, 100-seed weight, and yield of the common bean as affected by the long cropping seasons of years, bean varieties, cropping systems and their interactions.

Factors	Assessments	Measured variables in common bean				
		Yield (t ha^−1^)	Total biomass (t ha^−1^)	Pods per plant	Seeds per pod	100-seed wt (g)
Cropping systems (C)	Bean after maize (rotation)	1.8b	4.0	4.6	2.4	29.7
	Continuous bean (monoculture)	1.7a	3.9	4.8	2.2	29.3

Variety (V)	Improved bean *Lyamungu 90*	1.8	3.9	2.8a	2.3	32.3
	Local bean *Mkanamna*	1.6	4.1	6.5b	2.3	26.6

2-WAY-ANOVA (F-stat.)						
C		22.63 (*P* = 0.018)	0.01 (*P* = 0.939)	0.03 (*P* = 0.867)	0.2 (*P* = 0.688)	0.04 (*P* = 0.85)
V		3.54 (*P* = 0.109)	0.09 (*P* = 0.778)	15.76 (*P* = 0.007)	0.001 (*P* = 0.955)	1.81 (*P* = 0.228)
C × V		0.43 (*P* = 0.536)	0.001 (*P* = 0.974)	0.41 (*P* = 0.547)	0.02 (*P* = 0.894)	5.59 (*P* = 0.056)

Means with different letters differed significantly from each other. Interaction effects of cropping systems and bean varieties on total biomass test statistic W = 0.9603 (*P* = 0.667); Chi-square = 4.90 on 3^0^ of freedom (*P* = 0.179).

Multiple linear regression analysis results between bean grain yield and measured variables are presented in Table 6. Results indicated that 100-seed weight, total biomass and bean plant height had a positive influence on the increase in grain yield of beans.

**Table 6:** Estimates of parameters generated from multiple linear regression analysis based on a single short cropping season (2015) as their relationships with bean grain yield.

Parameter	estimate	s.e.	t(63)	t pr.
Constant (C)	1.718	0.633	2.71	0.03
100-seed weight (g)	0.0178	0.0118	1.51	0.174
Total biomass (t ha^−1^)	0.0397	0.0221	1.79	0.116
Pods per plant	−0.0089	0.0447	−0.2	0.847
Seeds per pod	−0.0377	0.0407	−0.93	0.385
Ground coverage at week 6	−0.124	0.169	−0.74	0.486
Ground coverage at week 8	0.124	0.164	0.76	0.474
Plant height at week 6	−0.0016	0.0172	−0.09	0.929
Plant height at week 8	−0.0054	0.0143	−0.38	0.717

The percentage variance accounted for is 40.0 and the standard error of observations is estimated to be 0.148. Weeks 6 and 8 represent 42 and 56 days, respectively after sowing.

### 3.3 Performance of maize in long rains

The main effect of long rainy seasons of cropping was significant on total biomass (P = 0.019) and 100-seed weight (P = 0.014) with the larger being 5.9 t ha^−1^ and 40.13 g, respectively in 2017 long season. The main effect of the cropping system was significant on maize grain yield (P = 0.039) and total biomass (P = 0.026). Also, interactions of both long rainy seasons and cropping systems were not significant on all measured variables in maize. The significantly larger maize grain yield (2.9 t ha^−1^) and total biomass (6.2 t ha^−1^) were obtained in maize sown as part of a rotation with the local bean variety Mkanamna as the main effect of cropping systems. There was no significant effect of the interactions between cropping seasons and cropping systems on the measured variables in maize during long rainy seasons of three years (Table 7).

**Table 7:** Grain yield, total biomass, and 100-seed weight of maize as affected by the long seasons of cropping years, cropping systems and their interactions for the measurements taken over three long cropping seasons (2015 to 2017).

Factors	Assessments	Measured variables in maize		
		Yield (t ha^−1^)	Total biomass (t ha^−1^)	100-seed wt (g)
Years of cropping (S)	2015 –Long rainy season	2.4	3.2a	28.45a
	2016 –Long rainy season	2.5	5.3ab	29.35a
	2017 –Long rainy season	2.2	5.9b	40.13b

Cropping systems (C)	M+L90AftM	2.0ab	4.1ab	32.24
	M+LbAftM	1.8a	3.6a	29.93
	M-Cont	2.3ab	4.6ab	31.69
	MAftL90	2.7ab	5.8ab	33.21
	MAftLb	2.9b	6.2b	34.66
2-WAY-ANOVA (F-stat.)				
S		0.52 (*P* = 0.619)	8.3 (*P* = 0.019)	9.42 (*P* = 0.014)
C		2.83 (*P* = 0.039)	3.15 (*P* = 0.026)	1.14 (*P* = 0.352)
S × C		1.15 (*P* = 0.355)	1.26 (*P* = 0.295)	2.0 (*P* = 0.074)

Means with different letters differed significantly from each other. *Key:* M ​+ ​L90AftM is maize intercropped with the improved bean variety *Lyamungu 90* sown after sole maize, M ​+ ​LbAftM is maize intercropped with the local bean variety *Mkanamna* sown after sole maize, M-cont is maize sown continuously (monoculture), MAftL90 is maize sown in rotation with improved bean variety *Lyamungu 90*, MAftLb is maize sown in rotation with local bean variety *Mkanamna*, and s.e.d. is the standard errors of differences of means. The main effect of cropping seasons on maize grain yield test statistic W = 0.9548 (*P* = 0.111) and Chi-square = 0.94 on 1^0^ of freedom (*P* = 0.333); the main effect of cropping seasons on total biomass test statistic W = 0.9594 (*P* = 0.160) and Chi-square = 0.19 on 1^0^ of freedom (*P* = 0.666); the main effect of cropping systems on 100-seed weight test statistic W = 0.9815 (*P* = 0.744); Chi-square = 6.10 on 4^0^ of freedom (*P* = 0.192).

Results of the multiple linear regression analysis between maize grain yield and measured variables from 2015 to 2017 long rainy seasons are presented in Table 8. Results indicated that the total biomass had significant (*P* < .001) influence on the increase in maize grain yield. Other important attributes of an increase in maize grain yield during long rainy seasons included maize plant height and ground coverage over time although the impact was not significant (Table 8).

**Table 8:** Estimates of parameters generated from multiple linear regression analysis based on three long cropping seasons (2016 and 2017) as their relationships with maize grain yield.

Parameter	estimate	s.e.	t(33)	t pr.
Constant (C)	−0.037	0.803	−0.05	0.964
Ground coverage (%) at week 6	−0.01634	0.00922	−1.77	0.082
Ground coverage (%) at week 8	0.0083	0.0123	0.68	0.502
Plant height (cm) at week 6	0.00576	0.00394	1.46	0.149
Plant height (cm) at week 8	0.0046	0.00375	1.23	0.225
Total biomass (t ha^−1^)	0.3452	0.0251	13.78	<.001
100-seed weight (g)	−0.00133	0.0092	−0.14	0.886

The percentage variance accounted for is 80.7 and the standard error of observations is estimated to be 0.438. Weeks 6 and 8 represent 42 and 56 days, respectively after sowing.

### 3.4 Performance of maize in short rains

The main effect of short rainy seasons was significant on maize grain yield (*P* = 0.007) and the total biomass (*P* = 0.03) but not on 100-seed weight. In the 2015 short rainy season, maize produced larger grain yield (2.6  t  ha^−1^) than grain yield (1.8  t  ha^−1^) produced in the 2016 short rainy season. However, the significantly larger total biomass (8.1  t  ha^−1^) was obtained in maize cultivated in the 2016 short rainy season. On the other hand, the main effect of cropping systems and its interactions with the seasons on all measured variables in 2015 and 2016 short rainy seasons were not significant. Results also indicated that interactions between short rainy seasons and cropping systems were not significant on all measured variables in maize (Table 9).

**Table 9:** Grain yield, total biomass, and 100-seed weight of maize as affected by the seasons, cropping systems and their interactions for the measurements taken over short rainy seasons of two years (2015 and 2016).

Factors	Assessments	Measured variables in maize		
		Yield (t ha^−1^)	Total biomass (t ha^−1^)	100-seed wt (g)
Years of cropping (S)	2015 –Short rainy season	2.6b	5.0a	34.92
	2016 –Short rainy season	1.8a	8.1b	32.2

Cropping systems (C)	Monoculture	1.6	6	33.28
	M-Aftm+Lb	1.8	5.6	33.59
	M-AftLb	1.9	6.1	34.61
	M-Aftm+L90	2	7	34.21
	M-AftL90	2.1	8	32.12
2-WAY-ANOVA (F-stat.)				
S		43.7 (*P* = 0.007)	15.04 (*P* = 0.03)	2.13 (*P* = 0.24)
C		0.34 (*P* = 0.847)	2.35 (*P* = 0.083)	0.33 (*P* = 0.855)
S × C		0.38 (*P* = 0.822)	0.78 (*P* = 0.547)	0.42 (*P* = 0.791)

Means with different letters differed significantly from each other. ***Key:*** M-Aftm+Lb is maize sown after the intercrop of maize and local bean variety *Mkanamna*, M-AftLb is maize sown after the local bean variety *Mkanamna*, M-Aftm+L90 is maize sown after the intercrop of maize and improved bean variety *Lyamungu 90*, and M-AftL90 is maize sown after the improved bean variety *Lyamungu 90*.

Results of the multiple linear regression analysis between maize grain yield and other measured variables in 2015 and 2016 short rainy seasons are presented in Table 10. Results indicated that the total biomass had positive and significant (*P* = 0.003) influence on the increase in maize grain yield during short rainy seasons of the two years.

**Table 10:** Estimates of parameters generated from multiple linear regression analysis based on two short rainy seasons of (2015 and 2016) as their relationships with maize grain yield.

Parameter	estimate	s.e.	t(33)	t pr.
Constant (C)	−2.08	1.31	−1.58	0.123
Ground coverage (%) at week 6	−0.0097	0.0176	−0.55	0.586
Ground coverage (%) at week 8	0.0452	0.0147	3.08	0.004
Plant height (cm) at week 6	0.0044	0.0106	0.41	0.683
Plant height (cm) at week 8	−0.00122	0.0062	−0.2	0.846
Total biomass (t ha^−1^)	0.1815	0.0566	3.21	0.003
100-seed weight (g)	0.0009	0.025	0.04	0.971

The percentage variance accounted for is 55.2 and the standard error of observations is estimated to be 0.699. Weeks 6 and 8 represent 42 and 56 days, respectively after sowing.

## 4 Discussion

### 4.1 Performance of common bean

The main effects of long rainy seasons, cropping systems, bean varieties, and their interactions significantly increased bean grain yields suggesting that these factors are important to be considered in the production of the common bean through rotation/intercropping with maize. The main effect of long rainy seasons contributed to the higher bean grain yield (3.3 t ha^−1^) in 2015 compared with other years. However, delay of rains in 2016 and 2017 long seasons could be one of the causes of low grain yields obtained in the bean. Further, sowing of the common bean as part of a continuous intercrop with maize produced higher bean grain yield (3.4 t ha^−1^) compared with bean sown as a monoculture and/or in rotations with maize where common bean started and ended in the cultivated land. The main effect of cropping systems was also realized on 100-seed weight (56.28 g) obtained in improved bean intercropped with maize in 2016. These main effects (on grain yield and 100-seed weight) have been observed after one year but two seasons of cropping (long and short in 2015) in which the same cropping systems (intercrops with maize) were always maintained on the same plots. The findings of the present study show that bean intercropped with maize was always higher in grain yield compared with bean sown in monoculture and/or in rotation with maize in all long rainy seasons. The higher performance of common bean in intercrops with maize could be attributed to complementarities between maize and common bean for growth resources including light, water, and nutrients [33].

In assessing the main effect of bean varieties, the local bean variety produced significantly larger grain yield (2.7 t ha^−1^) than the improved bean variety (1.6 t ha^−1^), which could be due to adaptability and escaping mechanisms of the local bean to harsh climatic conditions [33]. The local bean is also characterized by delayed growth during adverse conditions before sets for flower setting and/or rather delayed development, production of pods, smaller seed size and a more vigorous vine growth [13,33]. The local bean variety also produces massive leaves which created larger ground coverage before leaf senescence hence an improvement of soil health. Most of these leaves fall on the ground before bean plants are harvested and add organic residues and nutrients to the soil when decompose, which may benefit crops in the subsequent cropping season [33]. Besides, there was a lower incidence and severity of insect pests and diseases throughout the growing period for the local bean cropping systems compared with those systems where the improved bean was included [33,34]. There are other additional but not clearly distinguished ‘rotation effects', which are associated with rotations involving common bean as grain legumes on improving systems productivity [23,35,36]. These ‘other rotation’ effects, include improvement of soil physical and chemical properties, hastening of soil microbial activity, elimination of phytotoxic substances, application of growth-promoting (GP) substances and reduced disease incidence [37,38]. These ‘other rotation’ effects warrant further investigation as they are not assessed in the present study. Also, the higher performance of local bean than the improved bean substantiates the adaptability of the local bean to harsh climatic conditions and the realization of stable yield [15]. Further, the significant contribution of total biomass and the number of seeds per pod on bean grain yield over long rainy seasons is also justified in the present study by the multiple linear regression analysis between grain yield and the measured variables.

The interactions between long rainy seasons and cropping systems were significant on grain yield (4.4 t ha^−1^) in 2015 in bean intercropped with maize compared with bean sown as a monoculture (2.8 t ha^−1^) or as part of a rotation (2.7 t ha^−1^) with maize. This rotation is such that bean crop starts and ends in the long rainy season but the maize crop is included in the short rainy season, which is between the two long rainy seasons. Further, the importance of intercrops is observed in 2017 (that is the third long rainy season) in bean intercropped with maize where the larger grain yield (3.5 t ha^−1^) was recorded compared with grain yields obtained in bean monoculture (0.33 t ha^−1^) and bean rotated with maize (0.28 t ha^−1^) in the same year. These findings signify the importance of cropping seasons and the system by which bean is included in the maize-based cropping systems in a given long rainy season. Also, the findings depict that apart from considering long rainy seasons, it is also important to consider intercropping and/or rotational advantages of bean in maize-based systems over a monoculture bean.

The interactions between seasons and bean varieties were significant on grain yield (3.4 t ha^−1^) in improved bean in 2015 compared with the local bean variety and other long rainy seasons (2016 and 2017). However, in 2016 and 2017 long rainy seasons the grain yields were 2.9 and 1.9 t ha^−1^ respectively in the local bean, which was superior to those obtained in improved bean (0.7 and 0.9 t ha^−1^) in the same years. These findings provide an insight that better performance of improved bean is well observed at the beginning of experimentation but its continuous cultivation over time is negatively affected, probably, by variations in climatic factors including rains. On the other hand, the local bean seems to be stable in the production of better grain yield over time, which could be due to its adaptability and coupling mechanisms to harsh environments [33].

The effect of cropping systems and bean varieties interactions were significant on grain yield (4.6 t ha^−1^) in a local bean intercropped with maize compared with the improved bean (2.2 t ha^−1^) using the same cropping system. Rotational cropping where the bean crop starts and ends in seasons (such that maize is cropped between bean seasons) also resulted in a larger grain yield (1.8 t ha^−1^) in the local bean than the grain yield (1.2 t ha^−1^) in the improved bean. These findings suggest that the local bean is better suited to maize intercrops and/or rotations than the improved bean. This is probably due to trailing growth habit of escaping shading effect from tall maize and the ability to add more residues and nutrients to the soil as also indicted by Nassary et al. [33]. These findings provide an insight that growth characteristics of bean need to be well-known before the bean crop is included in maize-based cropping systems.

The effects of the interactions between long rainy seasons, cropping systems, and bean varieties were significant on bean grain yield (4.4 t ha^−1^) in intercrops of common bean with maize in 2015. Significantly larger 100-seed weight (40.25 g) was obtained in 2016 in a cropping system where the common bean was sown beginning in the rotation and also sown at the end of the cropping involved rotations with maize. Between 2015 and 2016 long rainy seasons is the 2015 short rainy season during which the sole maize was in the same plots. This finding suggests that the practice of including maize between two long rainy seasons of cropping common bean is an important option to increase the weight of seeds and hence the resulting grain yield in the bean. The performance of common bean crop assessed during the short rainy season (2015), which is preceded by a single cropped long rainy season (2015), provides varying insights about bean grain yield and other yield attributes. The main effect of cropping systems resulted in significantly larger grain yield (1.8 t ha^−1^) in a bean crop sown in rotation with maize compared with grain yield (1.7 t ha^−1^) obtained in a bean sown as a monoculture. The greater grain yield obtained in the bean is based on situations where bean crop is sown as part of a rotation with maize such that maize started on the same plots during the previous long rainy season (in 2015). This finding suggests that maize created a favourable environment where the subsequent bean crop was well suited for growth and production of better grain yield than yields in plots where the bean was continuously cultivated over successive cropping seasons.

Besides the fact that this is a short rainy season, crops in experimental fields were supplemented with irrigation and no disease and insect pests observed during the entire period of crop growth. Also, the main effect of bean varieties was significantly larger in the number of pods per bean plant (7) in the local bean compared with the pods (3) produced in the improved bean. This finding reflects some characteristics of the local bean of producing many leaves, pods, and seeds compared with the improved bean. The improved variety is highly affected by environmental conditions such as drought, excessive rains, and the outbreak of disease and insect pests although it is bred for high yielding [15]. Further analysis of the results through multiple linear regressions provides an insight that increases in grain yield of the bean during the short rainy season are largely determined by the height of a bean plant, 100-seed weight, and the total biomass of beans although the increase is not significant. This finding provides an important indication of the factors to be put into consideration to increase grain yield in common bean when are cultivated during short cropping seasons. Also, it is important to consider the outcomes related to the sowing of bean in rotation with maize (and which crop starts in a field) and/or sowing in a monoculture along with these factors.

### 4.2 Performance of maize

The main effect of long rainy seasons was significantly larger on maize total biomass (5.9 t ha^−1^) and 100-seed weight (40.13 g) in 2017 compared with total biomass yield produced in 2015 and 2016 long rainy seasons. This finding suggests that long rainy seasons are important in the increase in total biomass and weight of seeds in maize, which are also related to grain yield. Further, maize produced significantly larger grain yield (2.9 t ha^−1^) and total biomass (6.2 t ha^−1^) when sown as part of a rotation with the local bean as the main effect of cropping systems. Smaller maize grain yield (2.7 t ha^−1^) was obtained in the rotation of maize with improved bean, monoculture maize (2.3 t ha^−1^) and/or with other cropping systems (1.8 and 2.0 t ha^−1^) used in the present study. This finding reflects, probably, soil fertility improvement in situations where the bean is included in rotation with maize but much advantage is derived from the local bean, which might be through larger quantities of decomposed residues [13,33]. Further analysis through multiple linear regression indicated that a significant increase in maize grain yield in long rainy seasons (2015 to 2017) is dependent largely on the quantities of total biomass. This finding suggests that an increase in total biomass could result in grain yield advantages of maize over long rainy seasons. Also, there are other important reasons for an increase in maize grain yield including increased in maize plant height and the extent at which ground is covered by the crop over time of growth although the impact is not significant. Ojiem et al. [25] indicated that legumes increased maize grain yield when included as part of rotation compared with maize sown in a monoculture. These arguments are also supported by the importance of N_2_-fixing grain legumes in rotation with a non-fixing maize crop [13,20,39].

The increase in maize grain yields in rotations with the two contrasting bean varieties also depicts a rotational effect, which could not necessarily be due to benefits gained from residual N2-fixed but improvement in overall soil health/quality [23]. Previous studies have also indicated that other rotational benefits are derived from the improvement of soil properties and increase in mycorrhizal infection as well as shielding against disease and pests to the subsequent maize crop [[40], [41], [42]]. The findings of the present study are also consistent with studies conducted elsewhere [43,44]. Also, Kamanga [36] pointed that at least 50% of the N returned to the soil through the incorporation of dead and decomposed legume residues is sufficiently utilized by the subsequent cereal crop over the growing season.

Apart from the importance of common bean on N_2_-fixation for the subsequent maize crop, rainfall context is an important factor to consider. Thilakarathna et al. [45] indicated that rainfall variation is critical to the performance of common bean interventions on smallholder farmers. The inclusion of N2-fixing legumes as part of a rotation with maize is also indicated to be an important economic approach that provides farmers with an alternative of those most appropriate for their farms [46]. Also, the use of legumes in rotation with maize on smallholder farms reduces costs associated with the purchasing of N-containing fertilizers for the maize crop in the subsequent season [47]. In the present study, the main effect of cropping seasons produced significantly larger maize grain yield (2.6 t ha^−1^) in the 2015 short rainy season compared with maize grain yield (1.8 t ha^−1^) produced in 2016 short rainy season. The similar main effect of short cropping season produced significantly larger total biomass (8.1 t ha^−1^) in maize cultivated in 2016. The significantly larger maize grain yield obtained in the 2015 short season could be attributed to some rains experienced during that season compared with the 2016 short season which relied completely on supplemental irrigation of crops in the field. During these cropping seasons the shortage of rains could be the reason for lack of significant impact of cropping systems on the measured variables in maize including grain yield. Further analysis of the results through multiple linear regression indicated that increases in grain yield of maize during short rainy seasons are proportional to an increase of maize total biomass.

## 5 Conclusion

This study provides important drivers of intensification of maize and common bean rotations on smallholder farms to improve food security and generate income. Inclusion of intercrops (of both maize and common bean) as part of a rotation with one of these crops is an important element to intensify rotational cropping as they also overcome risks associated with food insecurity that could be caused by a complete failure of one crop in the season.

## Declaration of competing interest

The authors declare that the submitted work was carried out in the absence of any personal, professional, or financial relationships that could potentially be construed as a conflict of interest.
